# Prevalence, Patterns, Association with Biofilm Formation, Effects on Milk Quality and Risk Factors for Antibiotic Resistance of Staphylococci from Bulk-Tank Milk of Goat Herds

**DOI:** 10.3390/antibiotics10101225

**Published:** 2021-10-08

**Authors:** Daphne T. Lianou, Efthymia Petinaki, Peter J. Cripps, Dimitris A. Gougoulis, Charalambia K. Michael, Katerina Tsilipounidaki, Anargyros Skoulakis, Angeliki I. Katsafadou, Natalia G. C. Vasileiou, Themis Giannoulis, Eleni I. Katsarou, Chrysoula Voidarou, Vasia S. Mavrogianni, Mariangela Caroprese, George C. Fthenakis

**Affiliations:** 1 Veterinary Faculty, University of Thessaly, 43100 Karditsa, Greece; dlianou@vet.uth.gr (D.T.L.); peterjohncripps@gmail.com (P.J.C.); dgoug@vet.uth.gr (D.A.G.); cmichail@vet.uth.gr (C.K.M.); elekatsarou@vet.uth.gr (E.I.K.); vmavrog@vet.uth.gr (V.S.M.); 2 University Hospital of Larissa, 41110 Larissa, Greece; petinaki@med.uth.gr (E.P.); tsilipoukat@gmail.com (K.T.); skulakis@gmail.com (A.S.); 3 Faculty of Public and One Health, University of Thessaly, 43100 Karditsa, Greece; agkatsaf@vet.uth.gr; 4 Faculty of Animal Science, University of Thessaly, 41110 Larissa, Greece; vasileiounat@gmail.com (N.G.C.V.); themisgia@gmail.com (T.G.); 5 Department of Agriculture, University of Ioannina, 47132 Arta, Greece; xvoidarou@uoi.gr; 6 Department of Agriculture, Food, Natural Resources and Engineering (DAFNE), University of Foggia, 71122 Foggia, Italy; mariangela.caroprese@unifg.it

**Keywords:** bulk-tank milk, mastitis, methicillin, milk, goat, somatic cell counts, staphylococcus, tetracycline, total bacterial counts

## Abstract

The objectives of this work were to study the prevalence and the patterns of antibiotic resistance of staphylococcal isolates from bulk-tank milk of goat herds across Greece, to assess possible associations of the presence of antibiotic resistance with the quality of milk in these herds and to evaluate herd-related factors potentially associated with the presence of antibiotic resistance among these staphylococcal isolates. A cross-sectional study was performed on 119 goat herds in Greece. Bulk-tank milk samples were collected for bacteriological examination; staphylococcal isolates were evaluated for resistance to 20 antibiotics. Oxacillin-resistant, resistant to at least one antibiotic, and multi-resistant staphylococcal isolates were recovered from 5.0%, 30.3%, and 16.0% of herds, respectively. Of 80 isolates, 7.5% were resistant to oxacillin, 50.0% were resistant to at least one antibiotic and 27.5% were multi-resistant. Resistance was seen more frequently among coagulase-negative staphylococci (59.3%) than among *Staphylococcus aureus* (23.8%). Resistance was more frequent against penicillin and ampicillin (41.3% of isolates) and fosfomycin (27.5%). No association was found with biofilm formation by staphylococci. For recovery of oxacillin-resistant isolates, the presence of working staff in the herds emerged as a significant factor; respective factors for the isolation of staphylococci resistant to at least one antibiotic were part-time farming and high (>10) number of systemic disinfections in the farm annually. The same three factors concurrently were also identified to be significant for the recovery of multi-resistant isolates.

## 1. Introduction

In Greece, goat farming for milk production is a significant sector of the agricultural industry. Goat milk production in the country amounted to 143,270,500 L in 2019 [1], which accounts for 10% of European and 3% of world goat milk production [2]. The product is consumed as a drink or used in cheese manufacturing. Among the various cheese types produced, an important part is exported (e.g., ‘feta’ cheese), which indicates the international significance of the goat farming industry in the country.

The quality of raw milk is important because it contributes to the quality of cheese and is significant for public health. Among the various factors that account for the quality of raw goat milk, antibiotic-resistant bacteria are of prime importance.

Staphylococci are frequently recovered from bulk-tank milk of goat herds [3,4,5]. Most previous studies that examined staphylococcal isolates from bulk-tank milk of goat farms, evaluated mostly methicillin-resistance, with variable results. For example, in Pakistan, Altaf et al. [6] reported that 19% of 122 *S. aureus* recovered from the milk of goats showed resistance to methicillin, whilst Caruso et al. [7] reported that they recovered only one such isolate from bulk-tank milk of 66 goat farms in Italy. In research that evaluated more antibiotics, the proportion of resistant isolates was found to be up to 100% in Brazil [8] and Jordan [9]. Thus far, all studies related to the resistance of staphylococci from goat milk have focused on the patterns of resistance of the staphylococcal isolates; herd management factors that are potentially associated with the development of resistance have not been thoroughly studied.

The objectives of this work were (a) to study the prevalence and the patterns of antibiotic resistance of staphylococcal isolates from bulk-tank milk of goat herds across Greece, (b) to assess possible associations of the presence of antibiotic resistance with the quality of milk in these herds, and (c) to evaluate herd-related factors potentially associated with the presence of antibiotic resistance among these staphylococcal isolates.

## 2. Results

### 2.1. Staphylococcal Recovery and Presence of Antibiotic Resistance

In total, staphylococci were recovered from bulk-tank milk samples from 75 herds (63.0%, 95% CI: 54.1–71.2%). Of these, *Staphylococcus aureus* was isolated from samples from 21 (17.6%) herds and coagulase-negative staphylococci were isolated from samples from 54 (45.4%) herds. A total of 80 staphylococcal isolates were recovered (21 *S. aureus* and 59 coagulase-negative staphylococci) (Table 1).

Resistant (to at least one (any) antibiotic) or multi-resistant staphylococci were obtained from 36 (30.3%, 95% CI: 22.7–39.0%) or 19 (16.0%, 95% CI: 10.5–23.6%) herds, respectively. There was no difference in the proportion of farms in which resistant staphylococcal isolates were recovered according to their geographic part of the country, where they were located (*p* = 0.39) (Table 2).

Of the 80 staphylococcal isolates, 40 (50.0%, 95% CI: 39.3–60.7%) (5 *S. aureus* and 35 coagulase-negative isolates, *p* = 0.005 for comparison between *S. aureus* and coagulase-negative staphylococci; *p* = 0.026 for comparison between the various coagulase-negative species) were resistant to antibiotics. Further, 22 isolates (27.5%, 95% CI: 18.9–38.1%) (all coagulase-negative isolates, *p* = 0.001 for comparison between *S. aureus* and coagulase-negative staphylococci; *p* = 0.030 for comparison between the various coagulase-negative species) were multi-resistant. Details are presented in Table 1.

At isolate level, resistance was found more frequently against penicillin and ampicillin (33 isolates, 41.3% of all isolates), fosfomycin (22 isolates, 27.5% of all isolates), clindamycin (19 isolates, 23.8% of all isolates), erythromycin (16 isolates, 20.0% of all isolates), tetracycline (12 isolates, 15.0% of all isolates) and oxacillin (6 isolates, 7.5% of all isolates) (Table S1).

At herd level, staphylococci resistant to penicillin and ampicillin were isolated from 30 (25.2%, 95% CI: 18.3–33.7%) herds, to fosfomycin from 20 (16.8%, 95% CI: 11.2–24.5%), to clindamycin from 17 (14.3%, 95% CI: 9.1–21.7%), to erythromycin from 14 (11.8%, 95% CI: 7.1–18.8%), to tetracycline from 9 (7.6%, 95% CI: 4.0–13.8%), and to oxacillin from 6 (5.0%, 95% CI: 2.3–10.6%) herds.

Among the staphylococcal species, *S. aureus* was found to be resistant more frequently to penicillin (3/21 isolates), *S. equorum* was found to be resistant more frequently to ampicillin, erythromycin, penicillin, fosfomycin, and clindamycin (8/11, 8/11, 8/11, 7/11, and 6/11 isolates, respectively), and *S. capitis* to ampicillin, fosfomycin and penicillin (5/6 isolates for each antibiotic) (Table S1).

### 2.2. Biofilm Formation

Of the 80 isolates, 58 (72.5%, 95% CI: 61.9–81.1%) were found to be biofilm forming (Table 1). No association was seen between biofilm formation and resistance to antibiotics. Of the 40 resistant isolates, 28 (70.0%, 95% CI: 54.6–81.9%) (4 *S. aureus* and 24 coagulase-negative isolates) were biofilm forming (*p* = 0.62). Of the 22 multi-resistant isolates, 14 (63.6%, 95% CI: 43.0–80.3%) (all coagulase-negative isolates) were biofilm forming (*p* = 0.27). Further, no association was found with specific resistance to the antibiotics evaluated (*p* > 0.14 for all comparisons) (Table S2), as well as no association was found for specific staphylococcal species (*p* > 0.12 for all comparisons).

### 2.3. Associations with Milk Quality

Increased total bacterial counts (i.e., >1500 × 10^3^ cfu mL^−1^, which is the threshold set in the European Union for milk to undergo thermal processing [10]) were seen more commonly among herds from bulk-tank milk of which resistant staphylococcal isolates were recovered: 12/36 (33.3%) herds versus 17/83 (20.5%) herds without isolation of resistant staphylococci (*p* = 0.010). No other association of milk quality with the isolation of resistant staphylococci was found (Table S3).

### 2.4. Variables Associated with Isolation of Resistant or Multi-Resistant Staphylococcal Isolates from Bulk-Tank Milk

#### 2.4.1. Isolation of Oxacillin-Resistant Staphylococcal Isolates

During the univariable analysis, a significant association with isolation of oxacillin-resistant staphylococcal isolates from bulk-tank milk was evident for 2 of the 25 variables evaluated (Table S4). These were the following: education of the farmer and presence of working staff in the herd.

**Figure 1:**
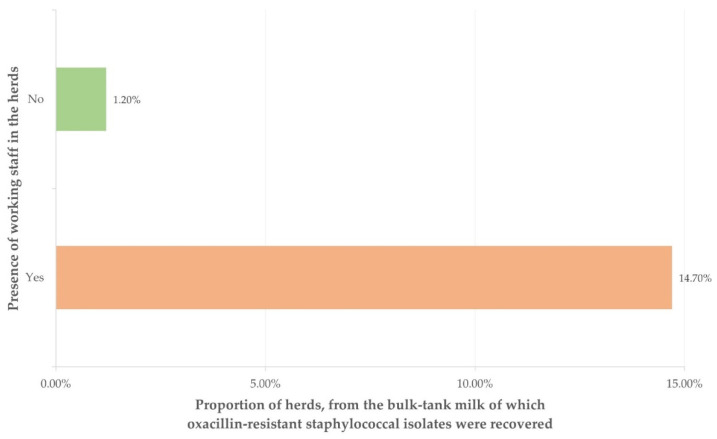
The proportion of herds from which oxacillin-resistant staphylococcal isolates were recovered, in terms of the presence of working staff in these herds.

Among the variables included in the multivariable analysis (Tables S4 and S5), only the following emerged as a significant factor: presence of working staff in the herd (Figure 1) (*p* = 0.005) (Table 3).

#### 2.4.2. Isolation of Staphylococcal Isolates Resistant to at Least One Antibiotic

During the univariable analysis, a significant association with isolation of resistant staphylococcal isolates from bulk-tank milk was evident for 6 of the 25 variables evaluated (Table S6). These were the following: management system applied in the herd, annual frequency of systemic disinfections in the farm, routine administration of antimicrobials in newborns, administration of ‘dry-ewe’ treatment at the end of the lactation period, farmer by profession, and presence of working staff in the herd.

**Figure 2:**
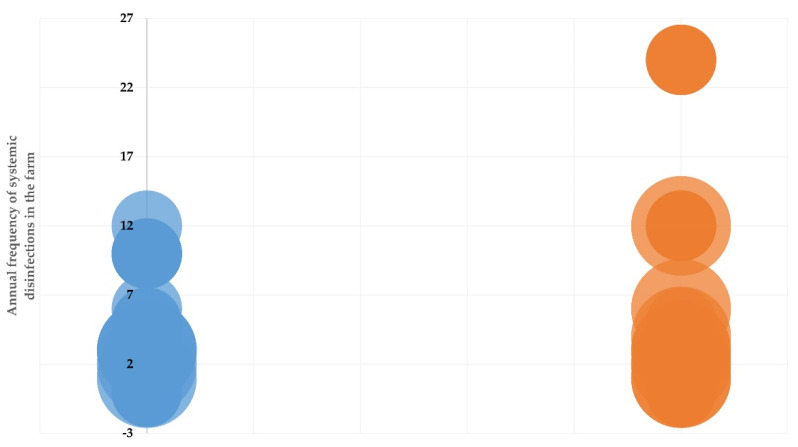
Frequency (shown by the circle diameter) of herds from which resistant staphylococcal isolates were (orange circles, *n* = 36) or were not (blue circles, *n* = 83) recovered from bulk-tank milk, in relation to the number of systemic disinfections performed annually in the herd (vertical axis; 0–1 occasions, *n* = 33, 2–10 occasions, *n* = 76, >10 occasions, *n* = 10) and the professional capacity of the farmer (solid pattern of circles: full-time farmers, *n* = 105; motif pattern of circles: part-time farmers, *n* = 14).

Among the variables included in the multivariable analysis (Tables S5 and S6), the following two emerged as significant factors: (a) farmer by profession (*p* = 0.001) and (b) annual frequency of systemic disinfections in the farm (*p* = 0.018) (Figure 2) (Table 4).

#### 2.4.3. Isolation of Multi-Resistant Staphylococcal Isolates

During the univariable analysis, a significant association with isolation of multi-resistant staphylococcal isolates from bulk-tank milk was evident for 3 of the 12 variables evaluated (Table S7). These were the following: annual frequency of systemic disinfections in the farm, farmer by profession, and presence of working staff in the herd.

Additionally, the same three emerged as significant among the variables included in the multivariable analysis (Tables S5 and S7): (a) annual frequency of systemic disinfections in the farm (*p* = 0.002), (b) presence of working staff in the herd (*p* = 0.016), and (c) farmer by profession (*p* = 0.022) (Table 5).

## 3. Discussion

The European Food Safety Authority has published a scientific opinion [11] that pointed out the public health significance of antibiotic resistance of bacteria isolated from raw milk. Hence, it is relevant to study the patterns of resistance in goat farms. Moreover, the evaluation and identification of predictors related to management could allow the implementation of procedures that help to limit the presence of antibiotic resistance in the farms.

This study included goat farms from all parts of Greece. Thus, conditions prevailing throughout the country were taken into account, and factors of regional importance weighed less. In order to minimise possible bias, the study also used consistent methodologies and ensured that specific tasks were always performed by the same investigators.

### 3.1. Presence of Antibiotic Resistance in Staphylococcal Isolates

In previous studies of caprine mastitis, *S. caprae*, *S. chromogenes*, *S. epidermidis*, *S. simulans*, *S. warneri*, and *S. xylosus* predominated as causal agents of the infection [12,13]. This indicates that many of the recovered isolates in the current study possibly originated from sources outside the animals. Apart from the milk of the goats (i.e., as agents of mastitis), the staphylococci could have originated from the skin udder and teat or from the equipment for milk handling and storage (e.g., teat cups, pipelines, milk tank) [14]. Further, in herds in which hand milking is applied, the staphylococci might have also originated from the hands of the milkers [15].

The extent of antibiotic resistance was, in general, similar to that presented in other relevant reports from the para-Mediterranean region where dairy goats are kept and milk is produced for human consumption. The results of this study showed low-level resistance among *S. aureus* isolates but a significantly greater problem among the coagulase-negative isolates. *S. aureus* is a significant causal agent of clinical mastitis in goats; it can be diagnosed easily and then followed by the initiation of appropriate treatment. In contrast, coagulase-negative isolates cause subclinical mastitis, an infection of lesser severity, which is difficult to diagnose and thus is treated infrequently. These organisms are also present in the environment or are part of a carrier state [16] in the animals; therefore, there are more opportunities for exposure to factors that lead to the development of resistance. These results are in line with those of a recent study that we performed on the antibiotic resistance patterns of ovine mastitis pathogens, where *S. aureus* isolates showed significantly less frequent resistance than the coagulase-negative isolates [17]. It is also possible that some of the coagulase-negative isolates originated from people (e.g., farm personnel), as some species (e.g., *S. hominis* or *S. haemolyticus*) are confirmed human pathogens. Moreover, the detection of resistance to fosfomycin, which is not licenced for veterinary use, further supports the suggestion that some of the recovered isolates likely were of human origin.

Limited associations were found between the recovery of resistant staphylococcal isolates from the milk and its quality. The increased total bacterial counts may also reflect a difficulty in treating cases of mastitis, due to the presence of resistant isolates [18] or also, possibly, the development of resistance by relevant bacteria in the farm. As total bacterial counts over the threshold of 1500 × 10^3^ cfu mL^−1^ could result in penalties in the milk price paid to farmers, the presence of resistant isolates would have tangible adverse consequences to farmers.

### 3.2. Predictors for Antibiotic-Resistant Staphylococcal Isolates

Three factors were found to be associated with the presence of resistance in the staphylococcal isolates recovered during the study. This suggests that there are many aspects of farm systems that can influence the development of antibiotic resistance.

Professional farmers have obviously appreciated the importance of preventing the development of antibiotic resistance in their herds. This had been repeatedly underlined in many relevant campaigns in Greece, staged by various public and private organisations within their areas of responsibility [19]. One such campaign was initiated by the Hellenic Veterinary Association [20] and involved the production of leaflets for farmers and the distribution to professionals to inform them about the significance of resistant bacterial isolates for the animals (e.g., increased animal morbidity and adverse financial effects) and the potential transmission to humans. Additionally, veterinarians would discuss with farmers and highlight the importance of preventing antibiotic resistance. We can thus postulate that part-time farmers were not fully aware of the importance of the problem and were following practices and procedures that promoted the development of resistance.

The increased number of disinfections performed in the herds was also identified as a significant predictor for the recovery of resistant isolates. The long-standing use of disinfectants has been found to lead to the development of resistance to these by staphylococcal isolates, especially given that many isolates bear genes specific for the development of resistance to these biocides (e.g., *qac*, which encodes for resistance to quaternary ammonium compounds [21]). The use of disinfectants in goat farms is related to cleaning and to the maintenance of biosecurity, aiming to protect animals and people against harmful biological agents. In piggeries also, the development of methicillin-resistance of staphylococcal isolates has been associated with increased use of disinfectants [22]. In dairy farms, this association is of particular concern, due to the necessity for increased and frequent use of disinfectants, as part of the routine for parlour cleaning post-milking. This also increases the chances for resistant bacterial isolates to enter into the chain of milk production, as was seen in this study. Cross-resistance of disinfectants with antibiotics has been shown in various bacteria [23,24], and wide use of benzalkonium-type disinfectants can promote antibiotic resistance due to co-selection. Further, according to some studies, there is a linkage between antibiotic and disinfectant resistance, which is either genetic (i.e., co-localisation of responsible genes in plasmid elements [25]) or functional [26], and the strains carrying both traits appear to have a strong selective advantage, due to their positive selection from the intense selective pressures, leading to the prevalence of multidrug resistance species. Various studies have revealed the genes and genetic networks responsible for the development of resistance mechanisms; their dissemination across strains and species is enhanced by specific mechanisms, while horizontal gene transfer figures are at the top of the list of the exchange of genetic elements. Resistance to antibiotics and disinfectants has been found to be highly associated in various bacterial species (e.g., resistance genes *qacF*, *qacΕΔ1*, *tet*, *sul* [27]), underlying the possibility that common mechanisms are governing the resistance mechanisms to multiple substances. Moreover, staphylococcal isolates can harbour multiple plasmids responsible for resistance to antibiotics, heavy metals, antiseptics, and disinfectants [28,29,30], which can explain the strong association between increased frequency of systemic disinfections and antibiotic resistance. Some of these genes can also encode for resistance to antibiotics, and it is possible that the use of disinfectants could lead to the elimination of susceptible isolates, thus contributing to the increased prevalence of multidrug-resistant isolates. These pose serious threats to both human and animal populations, leading to the development of alternative approaches to control bacterial growth [31], which will enhance the levels of biosecurity and the avoidance of threats for public health.

The presence of working staff in a farm was seen more often in herds with intensive management (78% of herds with intensive management in this study), where practices found to be associated with recovery of resistant isolates are often performed; working staff would be necessary for such time-consuming tasks, e.g., administration of antibiotics to newborns, frequent disinfections etc., etc. In their majority (in 32 of 36 herds, 89%; Lianou unpublished data) the working staff were of non-Greek ethnicity and possibly did not speak the local language well; therefore, one may postulate that they might not have fully assimilated the campaigns for preventing antibiotic resistance, thus following practices that might have contributed to that. Moreover, a study in the United States indicated that farmworkers could be healthy carriers of antibiotic-resistant bacterial strains [32]. Hence, they could have disseminated these within their farm or even to other farms if they changed their workplace.

## 4. Materials and Methods

### 4.1. Goat Herds and Sampling

**Figure 3:**
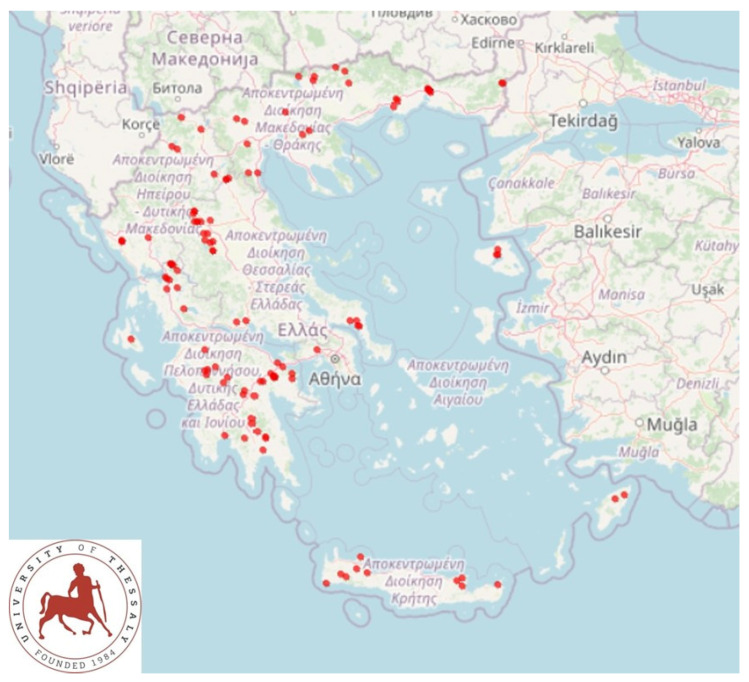
Location of 119 goat herds around Greece, which were visited for bulk-tank milk sampling.

A cross-sectional study involving 119 herds was performed from April 2019 to July 2020 and covered all the 13 administrative regions of Greece (Figure 3). Herds were included in the study on a convenience basis (willingness of goatherds to accept a visit by university personnel for interview and sample collection), as detailed before [5]. The principal investigators (D.T.L. and G.C.F.), accompanied by other investigators, visited all the herds for sample collection.

Initially, the management practices applied in the herds were recorded during an interview of the goatherd by means of a detailed questionnaire [33]. Bulk-tank milk samples were taken aseptically from each herd for somatic cell counting and milk composition evaluation and for bacteriological examinations. Samples were packed at 0.0 to 4.0 °C and transported for laboratory examinations [5].

### 4.2. Laboratory Examinations

Two milk samples from each bulk tank were used for somatic cell counts (SCC) and milk composition measurement; the other two were used for the bacteriological examinations. Two sub-samples were created and processed from each of the four samples so that each separate test was performed four times (each one in different sub-samples).

Somatic cell counting and milk content measurement were performed within 4 h of collection, whilst bacteriological examinations started within 24 h after collection of samples [Lianou et al. 2021]. Bacteriological examinations from each of the four relevant sub-samples included total bacterial counts (TBC), performed by employing the standardised procedures described by Laird et al. [34] and culturing on Staphylococcus selective medium (Mannitol salt agar; BioPrepare Microbiology, Athens, Greece) for aerobic incubation at 37 °C for 48 h; if there was no growth, media were reincubated for a further 24 h. After completion of sample aliquot withdrawal for microbiological examination, the temperature of the respective samples was measured and was never found to exceed 3.8 °C.

Bacterial isolation and initial identification by means of Gram staining and evaluation of catalase production were performed using standard methods [35,36]. Definite identification of the staphylococcal isolates to species level was performed by using matrix-assisted laser desorption/ionisation time-of-flight mass spectrometry (VITEK MS; BioMerieux, Marcy-l’-Étoile, France).

Then, in vitro biofilm formation by the staphylococcal isolates was evaluated. This was performed by using the combination of (a) the culture appearance on Congo Red agar plates and (b) the results of a microplate adhesion test. The procedures were detailed by Vasileiou et al. [37] for staphylococcal isolates recovered from milk.

Finally, the susceptibility testing to 20 antibiotics (amikacin, ampicillin, ceftaroline, ciprofloxacin, clindamycin, erythromycin, fosfomycin, fusidic acid, gentamicin, linezolid, moxifloxacin, mupirocin, mupirocin high level, oxacillin, penicillin G, rifampin, teicoplanin, tetracycline, tobramycin, trimethoprim–sulfamethoxazole) was performed by means of the automated system BD Phoenix™ M50 (BD Diagnostic Systems, Sparks, MD, USA). The interpretation of the results was based on the criteria of the European Committee on Antimicrobial Susceptibility Testing (EUCAST) (http://www.eucast.org (accessed on 17 September 2021)).

### 4.3. Data Management and Analysis

#### 4.3.1. Data Management

The presence of staphylococci in bulk-tank milk was defined by the isolation of ≥3 staphylococcal colonies on at least one agar plate of the four that were cultured with a sub-sample from each bulk-tank milk from a herd.

Biofilm formation by the staphylococcal isolates was indicated by a combination of the results of the two methods (culture appearance on Congo Red agar and microplate adhesion) [37] and staphylococcal strains were then characterised as biofilm-forming or non-biofilm-forming.

Based on the results of susceptibility/resistance testing, isolates were classified as susceptible, susceptible increased exposure, or resistant to each antibiotic according to the EUCAST criteria. As no ‘susceptible increased exposure’ isolates were found, this possible result was omitted from the analyses. Multidrug-resistant isolates were those found resistant to at least three different classes of antibiotics [38].

During cell counting, total bacterial counting, and milk content measurement for each bulk-tank milk sample, the results of the two sub-samples from each sample were averaged, and then the two means were again averaged for the final result regarding each bulk-tank milk.

SCCs were transformed to somatic cell scores (SCS) [39,40] by using the following formula: SCS = log_2_(SCC/100) + 3, and TBCs were transformed to log_10_; for both parameters, the transformed data were used in the analyses. For the presentation of results, the transformed findings were back-transformed as follows: 100 × 2^(SCS−3)^ for SCC and 10^log^ for TBC data.

#### 4.3.2. Statistical Analysis

Data were entered into Microsoft Excel and analysed using SPSS v. 21 (IBM Analytics, Armonk, NY, USA). Basic descriptive analysis was performed. Exact binomial confidence intervals (CI) were obtained.

The country was divided into four parts: central part, islands, northern part, and southern part, and herds were allocated to the appropriate one according to their geographical location. Pearson’s chi-squared test was employed to compare between the four parts of the country, the proportions of herds in each one, in which staphylococcal isolates were recovered.

In total, 25 variables were evaluated for potential association with the recovery of staphylococcal isolates resistant to antibiotics from bulk-tank milk of these herds (Appendix A); these were either taken directly from the answers of the interview performed at the start of the visit or calculated based on these answers. For each of these variables, categories were created according to the answers of the farmers.

The outcomes of ‘isolation of oxacillin-resistant staphylococcal isolates from bulk-tank milk’ and ‘isolation of resistant staphylococcal isolates from bulk-tank milk’ (i.e., isolates resistant to any (at least one) antibiotic) were considered. Exact binomial CIs were obtained. Initially, the importance of predictors was assessed by using cross-tabulation with Pearson’s chi-squared test and with simple logistic regression. Subsequently, multivariable models were created, initially offering to the model all variables, which achieved a significance of *p* < 0.2 in the univariable analysis. Variables were removed from the initial model by backward elimination. The *p* value of removal of a variable was assessed by the likelihood ratio test, and for those with a *p* value of >0.2, the variable with the largest probability was removed. This process was repeated until no variable could be removed with a *p* value of >0.2. The variables required for the final multivariable models are shown in Table S5.

Subsequently, the outcome of ‘isolation of multi-resistant staphylococcal isolates from bulk-tank milk’ was considered. Only the variables that achieved *p* < 0.2 in the analysis for isolation of resistant staphylococci were evaluated, and the same procedures as above (i.e., univariable and multivariable analyses) were performed. The variables required for the final multivariable model are shown in Table S5.

Finally, the potential association of isolation of a resistant staphylococcal isolate with SCC, TBC, and composition of bulk-tank milk was assessed by using a one-way analysis of variance.

In all analyses, statistical significance was defined at *p* ≤ 0.05.

## 5. Conclusions

The recovery of resistant staphylococci in bulk-tank milk in goat farms, which is intended for human consumption, raises concerns within the ‘one health’ concept. Potentially, the genetic material of these resistant staphylococci, which is not destroyed during the thermal processing of milk, might possibly be transferred to humans [41,42]. These genes could be incorporated into other bacteria, which constitute a part of the normal flora of humans. Thus, further dissemination of resistance genes can occur. Resistant staphylococcal isolates in raw milk from goats act as ‘containers’ of resistance genes and the dairy products as the means for their dissemination. This indicates the need for limiting the staphylococcal presence in the milk and for preventing resistance development in dairy goat herds, finally reducing the relevant public health concerns.

The current findings focused on identifying variables and factors in goat herds that can be related to the presence of resistant isolates in raw milk. These findings should act as a guide to allow the application of good practices, thus contributing to preventing the development of resistance and supporting the ‘one health’ concept.

As a future prospect, modern genetics and genomics techniques, such as multi-locus sequence typing (MLST) or whole-genome sequencing (WGS), can be applied, in order to study the origin of such resistant isolates (e.g., from people or other animals in the farms). Thus, further measures can be applied to minimise the risk of cross-species transmission events and to reduce the prevalence of staphylococcal species in milk and dairy products.

## Figures and Tables

**Table 1:** Frequency of staphylococcal species recovered from bulk-tank milk of 119 goat herds in Greece.

Staphylococcal Species	Frequency of Staphylococcal Isolates			
	All Isolates ^1^	Resistant Isolates ^2,3^	Multi-Resistant Isolates ^3^	Biofilm-Forming Isolates ^3^
*Staphylococcus aureus*	21 (0.263)	5 (0.238)	0 (0.000)	17 (0.810)
*Staphylococcus equorum*	11 (0.138)	9 (0.818)	8 (0.727)	8 (0.727)
*Staphylococcus simulans*	9 (0.113)	1 (0.111)	0 (0.000)	6 (0.667)
*Staphylococcus capitis*	6 (0.075)	5 (0.833)	2 (0.333)	5 (0.833)
*Staphylococcus lentus*	5 (0.063)	2 (0.400)	2 (0.400)	1 (0.200)
*Staphylcoccus haemolyticus*	4 (0.050)	1 (0.250)	0 (0.000)	2 (0.500)
*Staphylococcus vitulinus*	4 (0.050)	4 (1.000)	4 (1.000)	3 ().750)
*Staphylococcus kloosii*	3 (0.038)	2 (0.667)	2 (0.667)	2 (0.667)
*Staphylococcus pettenkoferi*	3 (0.038)	3 (1.000)	0 (0.000)	3 (1.000)
*Staphylococcus cohnii* subsp. *urealyticum*	2 (0.025)	1 (0.500)	1 (0.500)	1 (0.500)
*Staphylococcus lugdunensis*	2 (0.025)	2 (1.000)	0 (0.000)	1 (0.500)
*Staphylococcus warneri*	2 (0.025)	2 (1.000)	1 (0.500)	2 (1.000)
*Staphylococcus xylosus*	2 (0.025)	1 (0.500)	1 (0.500)	2 (1.000)
*Staphylococcus auricularis*	1 (0.012)	0 (0.000)	0 (0.000)	1 (1.000)
*Staphylococcus chromogenes*	1 (0.012)	0 (0.000)	0 (0.000)	1 (1.000)
*Staphylococcus cohnii* subsp. *cohnii*	1 (0.012)	1 (1.000)	1 (1.000)	0 (0.000)
*Staphylococcus epidermidis*	1 (0.012)	1 (1.000)	0 (0.000)	1 (1.000)
*Staphylococcus hominis*	1 (0.012)	0 (0.000)	0 (0.000)	1 (1.000)
*Staphylococcus intermedius*	1 (0.012)	0 (0.000)	0 (0.000)	1 (1.000)
Total	80	40 (0.500)	22 (0.275)	58 (0.725)

^1^ in brackets: proportion of isolates of the species among all isolates; ^2^ resistant to any (at least one) antibiotic; ^3^ in brackets: proportion of resistant, multi-resistant, or biofilm-forming isolates among the isolates of the respective species.

**Table 2:** Recovery of resistant staphylococcal isolates from bulk-tank milk of 119 goat herds in Greece, according to the part of the country from which the herds originated.

Location of Herds (Part of the Country)	Herds (*n*)	Herds in Which Resistant Staphylococcal Isolates Were Recovered (*n*) ^1^
Central part	36	13 (0.361)
Islands	16	2 (0.125)
Northern part	36	11 (0.306)
Southern part	31	10 (0.323)

^1^ in brackets: proportion of herds in which resistant staphylococcal isolates were recovered among all herds.

**Table 3:** Results of multivariable analysis for isolation of oxacillin-resistant staphylococcal isolates from bulk-tank milk of 119 goat herds in Greece.

Variable (*n* = 1)	Odds Ratio ^1^ (95% Confidence Intervals)	*p*
Presence of working staff in the herd		0.005
Yes (*n* = 34)	14.483 (1.624–129.171)	0.017
No (*n* = 85)	reference	-

^1^ odds ratio calculated against the lowest prevalence associations of variables.

**Table 4:** Results of multivariable analysis for isolation of resistant staphylococcal isolates from bulk-tank milk of 119 goat herds in Greece.

Variable (*n* = 2)	Odds Ratios ^1^ (95% Confidence Intervals)	*p*
Farmer by profession		0.001
Full-time (*n* = 105)	reference	-
Part-time (*n* = 14)	5.200 (1.602–16.882)	0.006
Annual frequency of systemic disinfectionsin the farm		0.018
0–1 occasion (*n* = 33)	reference	-
2–10 occasions (*n* = 76)	1.327 (0.499–3.529)	0.57
>10 occasions (*n* = 10)	33.429 (3.601–310.331)	0.002

^1^ odds ratio calculated against the lowest prevalence associations of variables.

**Table 5:** Results of multivariable analysis for isolation of multi-resistant staphylococcal isolates from bulk-tank milk of 119 goat herds in Greece.

Variable (*n* = 3)	Odds Ratio ^1^ (95% Confidence Intervals)	*p*
Annual frequency of systemic disinfectionsin the farm		0.002
0–1 occasion (*n* = 33)	reference	-
2–10 occasions (*n* = 76)	1.343 (0.339–5.317)	0.67
>10 occasions (*n* = 10)	23.333 (3.859–141.077)	0.0006
Presence of working staff in the herd		0.016
Yes (*n* = 34)	3.519 (1.281–9.668)	0.015
No (*n* = 85)	reference	-
Farmer by profession		0.022
Yes (*n* = 105)	reference	-
No (*n* = 14)	3.611 (1.056–12.349)	0.041

^1^ odds ratio calculated against the lowest prevalence associations of variables.

## Data Availability

Most data presented in this study are in the Supplementary Materials. The remaining data are available on request from the corresponding author. The data are not publicly available as they form part of the PhD thesis of the first author, which has not yet been examined, approved, and uploaded in the official depository of PhD theses from Greek Universities.

## Supplementary Materials

The following are available online at www.mdpi.com/article/10.3390/antibiotics10101225/s1, Table S1: Frequency of susceptibility/resistance to individual antibiotics of staphylococcal isolates recovered from bulk-tank milk of 119 goat herds in Greece, Table S2: Details of associations of antibiotic resistance with biofilm formation by staphylococcal isolates from bulk-tank milk of 119 goat herds in Greece, Table S3: Details of associations of milk quality with isolation of resistant or multi-resistant staphylococcal isolates from bulk-tank milk of 119 goat herds in Greece, Table S4: Results of univariable analysis for association with isolation of oxacillin-resistant staphylococcal isolates from bulk-tank milk of 119 goat herds in Greece, Table S5: Details of multivariable models employed for the evaluation of the isolation of resistant staphylococcal isolates from bulk-tank milk of 119 goat herds in Greece, Table S6: Results of univariable analysis for association with isolation of staphylococcal isolates resistant to at least one antibiotic from bulk-tank milk of 119 goat herds in Greece, Table S7: Results of univariable analysis for association with isolation of multi-resistant staphylococcal isolates from bulk-tank milk of 119 goat herds in Greece.

## Appendix A

**Table A1:** Variables (*n* = 25) evaluated for potential association with the presence of antibiotic resistance in staphylococci isolated from bulk-tank milk of 119 goat herds in Greece.

Management system applied in the herd (description according to EFSA classification) [EFSA 2014]
Month into the lactation period at sampling (month)
Machine- or hand-milking (description)
No. of does in the herd (no.)
Total milk quantity per doe obtained during the preceding milking period (litres)
Average number of kids born per doe (no.)
Collaboration with a veterinarian (yes/no)
Total visits made annually by veterinarians to the herd during the preceding season (no.)
Clinical mastitis annual incidence risk in the herd (%)
Age of kid removal from their dams (days)
Daily number of milking sessions (no.)
Duration of the dry period (months)
Means of calculating live bodyweight for the administration of pharmaceutical products (weighing/estimation)
Routine overdosing (compared to the dose prescribed) of pharmaceuticals (yes/no)
Annual frequency of systemic disinfections in the farm (no. of occasions)
Routine administration of antimicrobials in newborns (yes/no)
Vaccination against mastitis (yes/no)
Administration of ‘dry-ewe’ treatment at the end of the lactation period (yes/no)
Use of teat disinfection after milking (yes/no)
Age of the farmer (years)
Length of previous animal farming experience of the farmer (years)
Education of the farmer (description)
Farmer by profession (yes/no)
Family tradition in farming (yes/no)
Presence of working staff in the herd (yes/no)
