# Supplementary material for: Prevalence, Patterns, Association with Biofilm Formation, Effects on Milk Quality and Risk Factors for Antibiotic Resistance of Staphylococci from Bulk-Tank Milk of Goat Herds
Source: Antibiotics (Basel). 2021 Oct 8;10(10):1225. doi: 10.3390/antibiotics10101225 (PMC8532619; doi:10.3390/antibiotics10101225)
Supplement: Supplementary file 1 [file antibiotics-10-01225-s001.zip › antibiotics-1406102-supplementary.pdf]

Prevalence, Patterns, Association with Biofilm Formation, Effects on Milk Quality and Risk Factors for Antibiotic Resistance of Staphylococci from Bulk-Tank Milk of Goat Herds

Daphne T. Lianou, Efthymia Petinaki, Peter J. Cripps, Charalambia K. Michael, Dimitris A. Gougoulis, Katerina Tsilipounidaki, Anargyros Skoulakis, Angeliki I. Katsafadou, Natalia G.C. Vasileiou, Eleni I. Katsarou, Chrysoula Voidarou, Vasia S. Mavrogianni, Mariangela Caroprese and George C. Fthenakis

Table S1. Frequency of susceptibility / resistance to individual antibiotics [1] of staphylococcal isolates recovered from bulk-tank milk of 119 goat herds inGreece

	n	AMP	AZI	CXI	CIP	CLA	CLI	ERY	FOS	FUS	GEN	MOX	MUP	OXA	PEN	RIF	TEI	TET	TOB	SXT
S. aureus	21	3	0	0	0	0	1	0	0	0	0	0	0	1	3	0	0	2	1	0
S. equorum	11	8	0	0	0	0	6	8	7	0	0	0	0	1	8	0	0	2	0	0
S. simulans	9	1	0	0	0	0	0	0	0	0	0	0	0	0	1	0	0	0	0	0
S. capitis	6	5	0	0	0	0	1	0	5	0	0	0	0	0	5	0	0	2	0	0
S. lentus	5	2	0	0	0	0	2	0	0	1	0	0	0	2	2	0	0	1	0	0
S. haemolyticus	4	0	0	0	0	0	0	0	0	0	0	0	0	0	0	0	1	0	0	0
S. vitulinus	4	4	0	0	0	0	4	4	2	0	0	0	0	1	4	0	0	0	0	0
S. kloosii	3	2	0	0	0	0	2	1	1	1	0	0	0	1	2	0	0	1	0	0
S. pettenkoferi	3	1	0	0	0	0	0	1	3	0	0	0	0	0	1	0	0	0	0	0
S. cohnii ssp. *urealyticum*	2	1	0	0	0	0	1	0	1	1	0	0	0	0	1	0	0	0	0	0
S. lugdunensis	2	2	0	0	0	0	0	0	0	0	0	0	0	0	2	0	0	0	0	0
S. warneri	2	1	0	0	0	0	0	0	2	0	0	0	0	0	1	0	0	2	0	0
S. xylosus	2	1	0	0	0	0	1	1	0	0	0	0	0	0	1	0	0	0	0	0
S. auricularis	1	0	0	0	0	0	0	0	0	0	0	0	0	0	0	0	0	0	0	0
S. chromogenes	1	0	0	0	0	0	0	0	0	0	0	0	0	0	0	0	0	0	0	0
S. cohnii ssp. *cohnii*	1	1	0	0	0	0	1	1	1	0	0	0	0	0	1	0	0	1	0	0
S. epidermidis	1	1	0	0	0	0	0	0	0	0	0	0	0	0	1	0	0	1	0	0
S. hominis	1	0	0	0	0	0	0	0	0	0	0	0	0	0	0	0	0	0	0	0
S. intermedius	1	0	0	0	0	0	0	0	0	0	0	0	0	0	0	0	0	0	0	0
Total	80	33	0	0	0	0	19	16	22	3	0	0	0	6	33	0	1	12	1	0

[1] as established by use of VITEK 2; [2] AMP: ampicillin, AZI: azithromycin, CXI: cefoxitin, CIP: ciprofloxacin, CLA: clarithromycin, CLI: clindamycin, ERY: erythromycin, FOS: fosfomycin, FUS: fucidic acid, GEN: gentamicin, MOX: moxifloxacin, MUP: mupirocin, OXA: oxaxillin, PEN: penicillin, RIF: rifampicin, TEI: teicoplanin, TET: tetracycline, TOB: tobramycin, SXT: trimethoprim-sulfamethoxazole.

Table S2. Details of associations of antibiotic resistance with biofilm-formation by staphylococcal isolates from the bulk-tank milk of 119 goat herds in Greece.

**Antibiotic**	**Proportion of biofilm-forming resistant isolates**	***p***
All antibiotics	28/40 (70.0%)	0.62
Penicillin	23/33 (69.7%)	0.64
Ampicillin	21/31 (67.7%)	0.45
Fosfomycin	15/22 (68.2%)	0.59
Clindamycin	11/19 (57.9%)	0.14
Erythromycin	10/16 (62.5%)	0.32
Tetracycline	9/12 (75.0%)	0.83
Oxacillin	3/6 (50.0%)	0.20

Table S3. Details of associations of milk quality with isolation of resistant or multi-resistant staphylococcal isolates from the bulk-tank milk of 119 goat herds in Greece.

**Isolation of resistant staphylococcal isolates from the bulk-tank milk (*n* = 36)**	**No isolation of resistant staphylococcal isolates from the bulk-tank milk (*n* = 83)**	***p***
Somatic cell counts (cells mL^{-1})		
0.786×10^6 (95% CI: 0.652×10^6 - 0.948×10^6)	0.862×10^6 (95% CI: 0.764×10^6 - 0.967×10^6)	0.43
Herds with increased somatic cell counts (> 0.750×10^6 cells mL^{-1})		
22/36 (61.1%)	55/83 (66.3%)	0.78
Total bacterial counts (cfu mL^{-1})		
718×10^3 (95% CI: 468×10^3 - 1122×10^3)	530×10^3 (95% CI: 380×10^3 - 724×10^3)	0.28
Herds with increased total bacterial counts (> 1500×10^3 cfu mL^{-1})		
12/36 (33.3%)	17/83 (20.5%)	0.010
Fat (%)		
4.52 ± 0.17	4.88 ± 0.14	0.13
Protein (%)		
3.19 ± 0.03	3.24 ± 0.04	0.42
**Isolation of multi-resistant staphylococcal isolates from the bulk-tank milk (*n* = 19)**	**No isolation of multi-resistant staphylococcal isolates from the bulk-tank milk (*n* = 100)**	***p***
Somatic cell counts (cells mL^{-1})		
0.708×10^6 (95% CI: 0.560×10^6 - 0.897×10^6)	0.866×10^6 (95% CI: 0.775×10^6 - 0.967×10^6)	0.15
Herds with increased somatic cell counts (> 0.750×10^6 cells mL^{-1})		
10/19 (52.6%)	67/100 (67.0%)	0.23
Total bacterial counts (cfu mL^{-1})		
658×10^3 (95% CI: 513×10^3 - 851×10^3)	568×10^3 (95% CI: 436×10^3 - 741×10^3)	0.68
Herds with increased total bacterial counts (> 1500×10^3 cfu mL^{-1})		
6/19 (31.6%)	23/100 (23.0%)	0.42
Fat (%)		
4.59 ± 0.28	4.80 ± 0.12	0.49
Protein (%)		
3.27 ± 0.04	3.22 ± 0.03	0.54

Table S4. Results of univariable analysis for association with isolation of oxacillin-resistant staphylococcal isolates from the bulk-tank milk of 119 goat herds in Greece.

**Isolation of resistant staphylococcal isolates from the bulk-tank milk (n = 6)**				**No isolation of resistant staphylococcal isolates from the bulk-tank milk (n = 113)**				***p***
**Management system applied in the herd**								
Intensive	Semi-intensive	Semi-extensive	Extensive	Intensive	Semi-intensive	Semi-extensive	Extensive	
1	3	2	0	8	26	59	20	0.28
**Month into the lactation period at sampling**								
0–1st	2nd–5th	6th–9th	After 9th	0–1st	2nd–5th	6th–9th	After 9th	
0	3	3	0	8	57	40	8	0.76
**Machine- or hand-milking**								
Machine-milking	Hand-milking			Machine-milking	Hand-milking			
5	1			61	52			0.16
**No. of female goats in the herd**								
≤ 165 female goats	166-330 female goats	331-500 female goats	> 500 female goats	≤ 165 female goats	166-330 female goats	331-500 female goats	> 500 female goats	
3	2	0	1	53	35	13	12	0.82
**Total milk quantity per female goat obtained during the preceding milking period**								
≤ 200 L	201-400 L	> 400 L		≤ 200 L	201-400 L	> 400 L		
4	2	0		70	33	10		0.75
**Average number of kids born per female goat**								
≤ 1.50	> 1.50			≤ 1.50	> 1.50			
5	1			97	16			0.86
**Collaboration with a veterinarian**								
Yes	No			Yes	No			
5	1			96	17			0.91
**Total visits made annually by veterinarians to the herd during the preceding season**								
≤ 4	5 - 7	> 7		≤ 4	5 - 7	> 7		
3	1	2		48	24	41		0.93
**Clinical mastitis annual incidence risk in the herd**								
≤ 0.50%	> 0.50%			≤ 0.50%	> 0.50%			
1	5			47	66			0.23
**Age of kid removal from their dams**								
< 45 days	45–60 days	> 60 days		< 45 days	45–60 days	> 60 days		
0	5	1		26	39	48		0.05
**Daily number of milking sessions**								
1	2	3		1	2	3		
0	6	0		4	102	7		0.72

**Duration of the dry-period**						
≤ 2 months		> 2 months	≤ 2 months		> 2 months	
2		4	37		76	0.98
**Means of calculating live bodyweight for the administration of pharmaceutical products**						
Weighing		Estimation	Weighing		Estimation	
2		4	25		88	0.52
**Routine overdosing (compared to dose prescribed) of pharmaceuticals**						
Yes		No	Yes		No	
2		4	24		89	0.48
**Annual frequency of systemic disinfections in the farm**						
0 – 1 occasion	2- 10 occasions	> 10 occasions	0 – 1 occasion	2- 10 occasions	> 10 occasions	
3	3	0	30	73	10	0.40
**Routine administration of antimicrobials in newborns**						
Yes		No	Yes		No	
2		4	28		85	0.64
**Vaccination against mastitis**						
Yes		No	Yes		No	
2		4	33		80	0.83
**Administration of 'dry-ewe' treatment at the end of the lactation period**						
Yes		No	Yes		No	
1		5	14		99	0.76
**Use of teat disinfection after milking**						
Yes		No	Yes		No	
0		6	12		101	0.40
**Age of the farmer**						
Up to 50 years		Over 50 years	Up to 50 years		Over 50 years	
3		3	70		43	0.56
**Length of previous animal farming experience of the farmer**						
≤ 5 years		> 5 years	≤ 5 years		> 5 years	
2		4	22		91	0.41
**Education of the farmer**						
Primary education	Secondary or post-secondary education	Tertiary education	Primary education	Secondary education	Tertiary education	
1	2	3	19	87	7	0.0007

**Farmer by profession**				
Yes	No	Yes	No	
5	1	100	13	0.70
**Family tradition in farming**				
Yes	No	Yes	No	
5	1	99	14	0.76
**Presence of working staff in the herd**				
Yes	No	Yes	No	
5	1	29	84	0.002

Table S5. Details of multivariable models employed for the evaluation of the isolation of resistant staphylococcal isolates from the bulk-tank milk of 119 goat herds in Greece.

**Outcome**	Variables offered to the multivariable models (*n*)	**Variables required in the final models**
Isolation of an oxacillin-resistant staphylococcal isolate from the bulk-tank milk	4	(a) education of the farmer, (b) presence of working staff in the herd
Isolation of a resistant staphylococcal isolate from the bulk-tank milk	13	(a) clinical mastitis annual incidence risk in the herd, (b) frequency of systemic disinfections in the farm, (c) routine administration of antimicrobials in newborns, (d) length of previous animal farming experience of the farmer, (e) farmer by profession, (f) presence of working staff in the herd
Isolation of a multi-resistant staphylococcal isolate from the bulk-tank milk	7	(a) frequency of systemic disinfections in the farm, (b) routine administration of antimicrobials in newborns, (c) farmer by profession, (d) presence of working staff in the herd

Table S6. Results of univariable analysis for association with isolation of staphylococcal isolates resistant to at least one antibiotic from the bulk-tank milk of 119 goat herds in Greece.

**Isolation of resistant staphylococcal isolates from the bulk-tank milk (*n* = 36)**				**No isolation of resistant staphylococcal isolates from the bulk-tank milk (*n* = 83)**				***p***
**Management system applied in the herd**								
Intensive	Semi-intensive	Semi-extensive	Extensive	Intensive	Semi-intensive	Semi-extensive	Extensive	
6	12	15	3	3	17	46	17	0.015
**Month into the lactation period at sampling**								
0–1st	2nd–5th	6th–9th	After 9th	0–1st	2nd–5th	6th–9th	After 9th	
3	21	11	1	5	39	32	7	0.48
**Machine- or hand-milking**								
Machine-milking	Hand-milking			Machine-milking	Hand-milking			
23	13			43	40			0.22
**No. of female goats in the herd**								
≤ 165 female goats	166-330 female goats	331-500 female goats	> 500 female goats	≤ 165 female goats	166-330 female goats	331-500 female goats	> 500 female goats	
17	11	4	4	39	26	9	9	0.99
**Total milk quantity per female goat obtained during the preceding milking period**								
≤ 200 L	201-400 L	> 400 L		≤ 200 L	201-400 L	> 400 L		
22	11	3		52	24	7		0.98
**Average number of kids born per female goat**								
≤ 1.50	> 1.50			≤ 1.50	> 1.50			
30	6			72	11			0.62
**Collaboration with a veterinarian**								
Yes	No			Yes	No			
32	4			69	14			0.42
**Total visits made annually by veterinarians to the herd during the preceding season**								
≤ 4	5 - 7	> 7		≤ 4	5 - 7	> 7		
14	6	16		37	19	27		0.44
**Clinical mastitis annual incidence risk in the herd**								
≤ 0.50%	> 0.50%			≤ 0.50%	> 0.50%			
11	25			37	46			0.15
**Age of kid removal from their dams**								
< 45 days	45–60 days	> 60 days		< 45 days	45–60 days	> 60 days		
12	11	13		14	33	36		0.13
**Daily number of milking sessions**								
1	2	3		1	2	3		
0	33	3		4	75	4		0.32

**Duration of the dry-period**						
≤ 2 months	> 2 months		≤ 2 months	> 2 months		
16	20		23	60		0.07
**Means of calculating live bodyweight for the administration of pharmaceutical products**						
Weighing	Estimation		Weighing	Estimation		
6	30		21	62		0.30
**Routine overdosing (compared to dose prescribed) of pharmaceuticals**						
Yes	No		Yes	No		
7	29		19	64		0.68
**Annual frequency of systemic disinfections in the farm**						
0 – 1 occasion	2- 10 occasions	> 10 occasions	0 – 1 occasion	2- 10 occasions	> 10 occasions	
7	20	9	26	56	1	<0.0001
**Routine administration of antimicrobials in newborns**						
Yes	No		Yes	No		
14	22		16	67		0.024
**Vaccination against mastitis**						
Yes	No		Yes	No		
12	24		23	60		0.54
**Administration of 'dry-ewe' treatment at the end of the lactation period**						
Yes	No		Yes	No		
9	27		6	77		0.007
**Use of teat disinfection after milking**						
Yes	No		Yes	No		
6	30		6	77		0.12
**Age of the farmer**						
Up to 50 years	Over 50 years		Up to 50 years	Over 50 years		
24	12		49	34		0.43
**Length of previous animal farming experience of the farmer**						
≤ 5 years	> 5 years		≤ 5 years	> 5 years		
11	25		13	70		0.06
**Education of the farmer**						
Primary education	Secondary or post-secondary education	Tertiary education	Primary education	Secondary education	Tertiary education	
5	25	6	15	64	4	0.10-

**Farmer by profession**				
Yes	No	Yes	No	
27	9	78	5	0.003
**Family tradition in farming**				
Yes	No	Yes	No	
29	7	75	8	0.14
**Presence of working staff in the herd**				
Yes	No	Yes	No	
16	20	18	65	0.012

Table S7. Results of univariable analysis for association with isolation of multi-resistant staphylococcal isolates from the bulk-tank milk of 119 goat herds in Greece.

**Isolation of multi-resistant staphylococcal isolates from the bulk-tank milk (*n* = 19)**				**No isolation of multi-resistant staphylococcal isolates from the bulk-tank milk (*n* = 100)**				***p***
**Management system applied in the herd**								
Intensive	Semi-intensive	Semi-extensive	Extensive	Intensive	Semi-intensive	Semi-extensive	Extensive	
4	5	8	2	5	24	53	18	0.09
**C inical mastitis annual incidence risk in the herd**								
≤ 0.50%	> 0.50%			≤ 0.50%	> 0.50%			
6	13			42	58			0.40
**Duration of the dry-period**								
≤ 2 months	> 2 months			≤ 2 months	> 2 months			
8	11			32	68			0.39
**Annual frequency of systemic disinfections in the farm**								
0 – 1 occasion	2- 10 occasions	> 10 occasions		0 – 1 occasion	2- 10 occasions	> 10 occasions		
3	9	7		30	67	3		<0.0001
**Routine administration of antimicrobials in newborns**								
Yes	No			Yes	No			
8	11			22	78			0.06
**Administration of ‘dry-ewe’ treatment at the end of the lactation period**								
Yes	No			Yes	No			
4	15			11	89			0.23
**Use of teat disinfection after milking**								
Yes	No			Yes	No			
2	17			10	90			0.94
**Length of previous animal farming experience of the farmer**								
≤ 5 years	> 5 years			≤ 5 years	> 5 years			
6	13			18	82			0.18
**Education of the farmer**								
Primary education	Secondary and post-secondary education	Tertiary education		Primary education	Secondary and post-secondary education	Tertiary education		
3	12	4		17	77	6		0.09
**Farmer by profession**								
Yes	No			Yes	No			
14	5			91	9			0.030

**Family tradition in farming**				
Yes	No	Yes	No	
15	4	89	11	0.23
**Presence of working staff in the herd**				
Yes	No	Yes	No	
10	9	24	76	0.011
